# Life satisfaction and depressive symptoms of mentally active older adults in Poland: a cross-sectional study

**DOI:** 10.1186/s12877-021-02405-5

**Published:** 2021-08-18

**Authors:** Katarzyna Van Damme-Ostapowicz, Mateusz Cybulski, Monika Galczyk, Elżbieta Krajewska-Kulak, Marek Sobolewski, Anna Zalewska

**Affiliations:** 1 Department of Health and Caring Sciences, Faculty of Health and Social Sciences, Western Norway University of Applied Sciences, Førde, Norway; 2 Department of Integrated Medical Care, Faculty of Health Sciences, Medical University of Bialystok, Bialystok, Poland; 3 Department of Physiotherapy, Faculty of Health Sciences, Lomza State University of Applied Sciences, Lomza, Poland; 4 Department of Quantitative Methods, Faculty of Management, Rzeszow University of Technology, Rzeszow, Poland

**Keywords:** Older adults, Depression, Life satisfaction, Mental disorders, Mental sphere, Satisfaction with life, Satisfaction with life scale, Back depression inventory, Geriatric depression scale, Hospital anxiety and depression scale

## Abstract

**Background:**

For older adults, life satisfaction and depressive symptoms are related to quality of life. In this group of society, life satisfaction is particularly associated with the emotional area. The notion of life satisfaction is related to many factors, such as personality traits, moods and various life events, and poses challenges in various aspects of everyday life. Given that mental health is one of the determinants of the quality of life of older adults, it is reasonable to conduct research among this growing group of the population. The aim of this study was to assess life satisfaction and depressive symptoms in mentally active older adults in Poland.

**Methods:**

The study covered 125 attendees at the University of Healthy Senior (UHS) and 125 auditing students at the University of Psychogeriatric Prophylaxis (UPP), organised by the Faculty of Health Sciences at the Medical University of Bialystok, of whom 78.3% were female and 21.7% male. The study was conducted using four standardised scales: the Satisfaction with Life Scale (SWLS), Beck Depression Inventory, Geriatric Depression Scale (GDS), and Hospital Anxiety and Depression Scale (HADS).

**Results:**

Seniors who participated in the study were satisfied with their lives; the average SWLS score was 23 points. Men rated their level of satisfaction higher than women: the median score on the SWLS was 26 points for men and 23 points for women. Life satisfaction and mental disorders did not differ on the basis of sex, age, or education (the type of place of education attended). As the level of depression increased, life satisfaction decreased. Statistically significant correlations of average strength were found between the point values of the four measures of depression under consideration and were evenly distributed from 0.57 to 0.69.

**Conclusions:**

The high level of life satisfaction and a low level of mental disorders should be maintained in this population, and additional educational activities should be organised among seniors on a large scale. There were no differences in the distribution of psychometric measure scores among the three compared age groups of respondents in this study. Each of the questionnaires used measured of different aspects of depressive conditions, and it is worth using them in parallel rather than interchangeably.

## Background

In recent years, the issue of old age has become a subject of interest in scientific research due to the growth of the older adult population [1]. The literature points to important discoveries in this field, and differences have been noted regarding the impact on scientific literature on active ageing and older adult quality of life between European projects funded by the Seventh Framework Programme and similar US projects funded by the National Institutes of Health (NIH) [2, 3].

The concept of life satisfaction is useful for enhancing health, treatment and holistic care as well as for the rehabilitation process [4]. To assess the quality of and satisfaction with life, researchers in this area emphasise that the patient’s somatic condition, mental well-being, social relations and physical fitness should be taken into account [5**–**8]. Health condition is one of the basic factors of good quality of life and satisfaction with life [9]. Disease causes negative emotions and difficulties and forces people to limit or change the social functions they perform [10].

In older adults, quality of life is particularly associated with two areas: physical fitness, understood as health condition and vitality, and the cognitive and emotional area, which includes well-being and is broadly understood as life satisfaction [4]. The professional literature emphasises the benefits of physical activity as a means of preventing depressive symptoms in older adults [11]. Recent research by Harvey et al. indicated that regular free-time exercise of any intensity offers protection against future depression and that relatively small changes in the level of physical activity in the population may have significant benefits for mental health and prevent a significant number of new cases of depression [12].

Similar research results indicating the benefits of physical activity in older adults were shown in studies by Aguilar-Latorre et al. [13], Härkänen et al. [14], and Kim et al. [15].

The period of late adulthood is often associated with the occurrence of unfavourable life changes resulting in feelings of uselessness, loneliness, and helplessness; a lack of satisfaction; and, consequently, social withdrawal and other depressive symptoms [16–18]. It is estimated that depressive disorders affect approximately 20% of people over 65 years of age and are most often chronic [19–21]. At the beginning of the twenty-first century, depressive disorders in the United States were present in approximately 20% of the population over 55 years of age. However, global statistics show that this problem has increased in the last few years in most countries [22]. These disorders manifest primarily in a decline in activity or depression, which leads to problems in everyday life and disability [23–25].

Therefore, in light of the considerations presented above, it seems justified to undertake research to evaluate life satisfaction and the occurrence of mental disorders (the severity of nonpsychotic symptoms of mental functions and depressive symptoms) among older adults (in this study, mentally active people living in the Voivodeship of Podlaskie in Poland) through subjective evaluation to search for theoretical dependencies, to develop practical postulates in this field and to compare the results with those from different parts of Poland and other countries.

The research question in this study was as follows: Does regular mental activity undertaken by older adults help to prevent the decrease in life satisfaction and increase in depressive mood commonly observed with the ageing process?

We assumed that although the surveyed group consisted of socially, physically and mentally active people with healthy and active ageing, this group would be characterised by certain mental disorders and that the respondents’ subjective evaluation of life satisfaction would be at a low level.

## Methods

### Study design and participants

A quantitative cross-sectional research design was implemented for this study. The study was conducted among a group of mentally active older adults (auditing students at universities) living in the Voivodeship of Podlaskie: 125 participants from the University of Healthy Senior (UHS) and 125 auditing students from the University of Psychogeriatric Prophylaxis (UPP), organised by the Faculty of Health Sciences at the Medical University of Bialystok (Poland), in the period from January 2019 to May 2019.

The criteria for inclusion in the study group were as follows: 1) age ≥ 60, 2) residence in the Voivodeship of Podlaskie, 3) no recognised dementia or mental impairment (information about their cognitive functioning was indicated through evidence provided by the Clock-Drawing Test), 4) the ability to write and read independently, and 5) the provision of written consent to participate in the study.

The criteria for exclusion from the study group were as follows: 1) age below 60, 2) residence in a voivodeship other than Podlaskie, 3) a diagnosis of dementia or mental disability, 4) an inability to write and read independently, and 5) a lack of written consent to participate in the study.

Of the 250 questionnaires distributed among the volunteers willing to participate in the study, 92 questionnaires met the criteria for inclusion in the study.

In total, 72 women (78.3%) and 20 men (21.7%) took part in the study. The age of the surveyed seniors ranged from 60 to 75 or more, and the average age of the respondents was 67 ± 4 years. The most numerous categories consisted of respondents aged 60–65 and up to 70. Just over half of the respondents were married, 27.2% had lost their spouse, and one in 10 respondents was divorced. The vast majority of seniors (76%) were residents of towns in the Voivodeship of Podlaskie, one-sixth lived in a large city, and less than one-tenth of respondents lived in a village. In the surveyed population, one-third of the respondents had higher education, 41% had graduated from high school, and one-fifth of respondents did not provide information about their level of education. The persons surveyed attended classes offered by the University of Healthy Senior (65.2%) or the University of Psychogeriatric Prophylaxis (34.8%).

The respondents’ detailed sociodemographic data characteristics are shown in Table 1.

**Table 1:** Sociodemographic data of the surveyed older adults

Feature	Category	***N***	%
**Sex**	female	72	78.3
	male	20	21.7
**Age [years]**	60–65	32	34.8
	66–70	30	32.6
	71–75	18	19.6
	> 75	12	13.0
**Place of residence**	voivodeship city	70	76.1
	city	15	16.3
	town	1	1.1
	village	6	6.5
**Education**	Higher	31	33.7
	secondary	38	41.3
	vocational	4	4.3
	Primary	1	1.1
	no data available	18	19.6
**Financial standing**	very good	7	7.6
	good	48	52.1
	average	34	37.0
	rather poor	1	1.1
	poor	2	2.2
**Marital status**	married	51	55.4
	widowed	25	27.2
	single	4	4.3
	divorced	10	10.9
	separated	1	1.1
	no answer	1	1.1
**Family structure**	one generation	34	37.0
	three generations	22	23.9
	multi-generation	7	7.6
	two generations	25	27.2
	no answer	4	4.3
**Place of education**	University of Psychogeriatric Prophylaxis	32	34.8
	University of Healthy Senior	60	65.2

### Procedural and ethical considerations

The selection of participants was intentional, i.e., the authors of the study deliberately selected a group of mentally active older adults (auditing students of the universities) to determine the occurrence of symptoms of mental disorders and to assess life satisfaction.

Respondents were informed about the objective and hypotheses of the study, the voluntary nature of participation in the study and the possibility to withdraw from the study at any stage. Each participant was asked to provide written consent to participate in the study and was free to withdraw from the study at any stage.

A total of 250 questionnaires were distributed since so many participants volunteered to participate in the study; however, not all of them were returned to the authors of the study, and not all volunteers met the inclusion criteria.

After receiving the returned questionnaires, the authors determined that 92 completed survey questionnaires qualified for inclusion in the study.

The respondents received paper copies of the questionnaires to be filled out at home. The members of the research team informed and trained the participants in detail about how to correctly fill out the questionnaires. The completed questionnaires were returned by the study participants before the next lectures at their university.

The research conformed with the Good Clinical Practice guidelines, and the procedures were in accordance with the principles of the 1975 Declaration of Helsinki as revised in 2000 and with the ethical standards of the institutional committee on human experimentation. The study obtained ethical approval from the Bioethics Committee of the Medical University in Bialystok, Poland (R-I-002/144/2016).

### Measurements

The following four standardised assessment instruments were used to measure life satisfaction and the incidence of mental disorders in mentally active older adults. Numerical values calculated based on the standardised Satisfaction with Life Scale (SWLS), Hospital Anxiety and Depression Scale (HADS), Beck Depression Inventory (BDI) and Geriatric Depression Scale (GDS) questionnaires constituted the basis for classifying the respondents in one of the levels in terms of life satisfaction, depression or anxiety.

#### SWLS – Satisfaction with Life Scale

The Satisfaction with Life Scale (SWLS) was developed by E. Diener, R.A. Emmons, R.J. Larson, and S. Griffin [26], with a Polish adaptation by Z. Juczyński [27]. The SWLS consists of five statements for which respondents assess the extent to which each question relates to their life on the following scale: “completely disagree” – 1, “disagree” – 2, “somewhat disagree” – 3, “neither agree nor disagree” – 4, “somewhat agree – 5”, “agree” – 6, and “completely agree” – 7. The result is a general indicator of the sense of life satisfaction. The higher the score, the higher the sense of life satisfaction. The following scores indicate the levels of satisfaction with life: 31–35 – extremely satisfied; 26–30 – satisfied; 21–25 – slightly satisfied; 20 – neutral; 15–19 – slightly dissatisfied; 10–14 – dissatisfied; 5–9 – extremely dissatisfied [26, 27].

#### Beck Depression Inventory

The Beck Depression Inventory (BDI), which is a self-report questionnaire, was developed in 1961 by Beck (1961) [28] and adapted by Parnowski and Jernajczyk [29]. The BDI is used for the self-assessment of the presence and severity of depressive symptoms. Due to its simplicity and effectiveness, it is one of the most frequently used psychological tests. The BDI takes approximately 10 min to complete. For the test to have diagnostic value, the respondent should choose the answer that is closest to the “average” value during the past 30 days. The BDI consists of 21 items rated between 0 and 3, depending on the intensity of the symptoms. For each item, the respondent chooses one answer that, in the respondent’s opinion, best describes his or her condition during the indicated time period (before the doctor asks the patient to fill in the scale, the appropriate period of time should be specified – month, week or last day).

For people with diagnosed depression, a result of 0 to 9 points indicates the least severe symptoms of depression, 10 to 16 points indicates mild depression, 17 to 29 points indicates moderate depression, and 30 to 63 points indicates severe depression [30]. For the original version, Cronbach’s alpha coefficients were 0.27 to 0.74 in the control group and 0.39 to 0.70 in the group with depressive disorders; for the whole scale, Cronbach’s alpha coefficients were 0.93 and 0.92, respectively [31].

#### Geriatric Depression Scale

The full version of the Geriatric Depression Scale (GDS) is often used in geriatric wards to assess patients’ emotional condition [32, 33]. The GDS was developed in 1983 by Yesevage et al. [32]. The scale is a screening tool consisting of 30 questions. When performing the self-assessment, the patient answers by marking “yes” or “no”. The GDS is a self-assessment scale, and help in reading and completing the scale for respondents with weakened intellectual functions is acceptable. The interpretation of the full version is as follows: 0–10 points indicates no depression, 11–20 indicates mild depression, and > 21 indicates deep depression. Each response is scored 0 or 1 point; hence, the overall score for the full version can range between 0 and 30 points. Scores from 0 to 9 points correspond to a lack of depression, whereas those from 10 to 19 points and from 20 to 30 points correspond to mild and severe depression, respectively.

The scale is characterised by high consistency and reliability in patients with depression in the course of Alzheimer’s disease; the GDS is the most commonly used scale in assessing depression in old age [32, 33].

#### Hospital Anxiety and Depression Scale

The original version of the Hospital Anxiety and Depression Scale (HADS) was developed by A. Zigmond and R. Snaith in 1983 to screen the level of anxiety and depression in patients treated in hospitals [34]. The questionnaire consists of seven questions assessing the symptoms of depression, especially pleasure disorders, and the most important seven questions from the Clinical Scale of Anxiety developed by R. Snaith. The result of the test is the sum of points in a given category. The Polish version of the test was developed and modified by M. Majkowicz, K. de Walden-Gałuszko, and G. Chojnacka-Szawłowska [35, 36]. In addition to the scales evaluating depression and anxiety, the questionnaire was supplemented with two questions assessing irritation and aggression.

The range of possible points is 0–21, with a higher result indicating higher severity of anxiety symptoms. A score within the normal range is 0–7 points; 8–10 is a borderline score, indicating the possible occurrence of severe anxiety; and a score of 11 or above is considered a pathological score, indicating the likelihood of anxiety disorders [35, 36].

### Statistical analysis

The distribution of psychometric measure scores was described using selected descriptive statistics (e.g., the mean, median, standard deviation).

The Mann-Whitney or Kruskal-Wallis test was used to assess the significance of differences in the distribution of life satisfaction and depression scores between groups of respondents with different demographic characteristics. The choice of nonparametric tests was dictated by a significant right-handed asymmetry of the distribution of some scores (especially HADS and BDI scores).

For the same reason, the nonparametric Spearman rank correlation coefficient was used to assess the correlations between the assessments of the mental condition of the respondents made using various psychometric measures.

Regression analysis was used to assess the association of demographic and social factors with the mental condition of the respondents in more detail. Two regression models were constructed to explain the variability of the SWLS and GDS scores in the surveyed population.

The selected results were illustrated with scatter diagrams or graphs of mean psychometric measure scores in the compared groups to illustrate statistically significant interactions occurring in the developed regression models.

For the differences and correlations, *p* < 0.05 was considered statistically significant. For better readability of the results, statistically significant effects for *p* < 0.05, *p* < 0.01 and *p* < 0.001 are marked with appropriate symbols (*, **, and ***, respectively) in the tables.

## Results

### Assessments of the mental condition of ***older adults***

Based on the information obtained from the SWLS, BDI and HADS questionnaires, the scores on the psychometric measures were determined for each respondent to assess the respondents’ satisfaction with life and the intensity of negative emotions (mainly related to the occurrence of depressive conditions). The distribution of these scores in the surveyed population is presented in Table 2. The average SWLS score was 23.0 points, the average BDI score was 8.1 points, the average HADS-A score was 5.5 points, the average HADS-D score was 3.9 points, and the average GDS score was 8.2 points.

**Table 2:** Characteristics of the distribution of psychometric measures in the surveyed community

Psychometric measures	Mean	Median	SD	Lower quartile	Upper quartile	Min	Max
SWLS	23.0	23	5.7	20	27	6	35
BDI	8.1	6	6.8	3	11	0	35
HADS-A (anxiety)	5.5	5	3.7	3	8	0	15
HADS-D (depression)	3.9	3	3.4	1	6	0	14
GDS	8.2	7	6.2	4	12	0	24

The results of the classification of the psychometric measure scores according to the standards are presented in Table 3.

**Table 3:** Classification of the values of psychometric measures according to the standards

Psychometric measures	Adjectival scale (points range)	***N***	%
Satisfaction with life (SWLS)	definitely unsatisfied (5–9)	1	1.1
	very unsatisfied (10–14)	7	7.8
	rather unsatisfied (15–19)	13	14.4
	indifferent (20)	5	5.6
	rather satisfied (21–25)	31	34.4
	very satisfied (26–30)	26	28.9
	definitely satisfied (31–35)	7	7.8
Severity of depression (BDI)	none (0–26)	69	75.8
	moderate/severe (27–63)	22	24.2
Anxiety level (HADS-A)	normal (0–7)	67	73.6
	borderline (8–10)	14	15.4
	abnormal (11–21)	10	11.0
Depression level (HADS-D)	normal (0–7)	79	86.8
	borderline (8–10)	6	6.6
	abnormal (11–21)	6	6.6
Depression level (GDS)	none (0–10)	64	71.1
	slight/deep (11–30)	26	28.9

### Factors associated with the mental condition of ***older adults***

A comparative analysis of the mental condition of older adults was performed in groups distinguished by sex, age, place of education, financial standing and marital status. To substantiate the results of the analyses performed, some groups were merged; for example, marital status was analysed in a dichotomous division of married and single people. In Tables 4, 5, 6, 7 and 8, descriptive statistics of the psychometric measure scores in the compared groups are presented, along with the significance of differences assessed using the Mann-Whitney or Kruskal-Wallis test.

**Table 4:** Sex vs. mental condition of older adults

Psychometric measures	Sex						***p***
	Female			Male			
	Mean	Median	SD	Mean	Median	SD	
SWLS	22.44	23	5.30	25.00	26	6.86	0.0734
BDI	8.27	6	7.00	7.30	7	5.87	0.6931
HADS-A	5.80	5	3.77	4.40	4	3.20	0.1370
HADS-D	3.68	3	3.38	4.60	4	3.23	0.1891
GDS	8.14	6	6.32	8.37	8	5.71	0.6307

*p*-value was calculated using Mann-Whitney test

**Table 5:** Age vs. mental condition of respondents

Psychometric measures	Age									***p***
	60–65 years			66–70 years			71–75 years			
	Mean	Median	SD	Mean	Median	SD	Mean	Median	SD	
SWLS	22.48	25	6.69	22.31	23	5.37	24.13	23	4.93	0.5182
BDI	8.48	7	6.79	6.97	5	6.05	8.70	6.5	7.44	0.4130
HADS-A	5.94	5	3.87	5.90	5	3.32	4.63	4	3.80	0.1785
HADS-D	3.68	4	3.21	3.80	3	3.45	4.17	3.5	3.49	0.8541
GDS	8.29	6	6.44	8.52	7	6.14	7.77	7	6.09	0.8892

*p*-value was calculated using Kruskal-Wallis test

**Table 6:** Place of education vs. mental condition of respondents

Psychometric measures	School						***p***
	University of Psychogeriatric Prophylaxis			University of Healthy Senior			
	Mean	Median	SD	Mean	Median	SD	
SWLS	22.13	23.5	5.91	23.45	23	5.61	0.3896
BDI	9.47	8.5	7.65	7.29	6	6.14	0.2122
HADS-A	6.06	5	4.38	5.19	4	3.25	0.4897
HADS-D	4.34	4	3.41	3.63	3	3.32	0.2797
GDS	8.78	6.5	7.00	7.86	7	5.69	0.8177

*p*-value was calculated using Mann-Whitney test

**Table 7:** Financial standing vs. mental condition of respondents

Psychometric measures	Financial standing						***p***
	Good			average			
	Mean	Median	SD	Mean	Median	SD	
SWLS	24.95	25	4.60	19.89	20	5.99	0.0001***
BDI	7.02	6	5.07	9.64	7	8.56	0.3101
HADS-A	4.96	4	3.54	6.31	5.5	3.80	0.0478*
HADS-D	3.13	2	2.80	5.03	4	3.82	0.0174*
GDS	6.76	5	5.20	10.43	10	6.94	0.0125*

*p-*value was calculated using Mann-Whitney test

* *p* < 0.05, *** *p* < 0.001

**Table 8:** Marital status vs. mental condition of respondents

Psychometric measures	Marital status						***p***
	married			single			
	Mean	Median	SD	Mean	Median	SD	
SWLS	24.04	25	6.29	21.87	21	4.65	0.0263*
BDI	8.16	6	7.10	7.67	6	6.25	0.7496
HADS-A	5.68	5	3.50	5.18	4	3.93	0.3216
HADS-D	4.04	3.5	3.54	3.50	3	2.95	0.5743
GDS	8.16	7	5.92	8.10	6.5	6.55	0.6606

*p*-value was calculated using Mann-Whitney test

* *p* < 0.05

Older men were characterised by an SWLS score that was slightly higher than that for older women (25.0 vs. 22.4 points); this difference was close to statistically significant (*p* = 0.0734). Depression and anxiety measure scores did not differ on the basis of sex. In addition, the mental condition of older adults did not differ on the basis of age or the place of education. People with relatively worse financial standing had lower life satisfaction and a higher level of anxiety or depression (statistically significant differences for all analysed measure scores except for the BDI scores). Married people had significantly higher life satisfaction than single people (24.0 vs. 21.9 points).

### Correlations between psychometric measure scores

In Table 9, the values of Spearman’s rank correlation coefficients for the SWLS, BDI, HADS and GDS scores are presented.

**Table 9:** Correlations between psychometric measures

Psychometric measures	SWLS	BDI	HADS-A (anxiety)	HADS-D (depression)	GDS
**SWLS**	1	−0.45	−0.36	−0.41	−0.54
**BDI**	−0.45	1	0.57	0.63	0.69
**HADS-A (anxiety)**	−0.36	0.57	1	0.61	0.62
**HADS-D (depression)**	−0.41	0.63	0.61	1	0.67
**GDS**	−0.54	0.69	0.62	0.67	1

*** *p* < 0.001

**Fig. 1:**
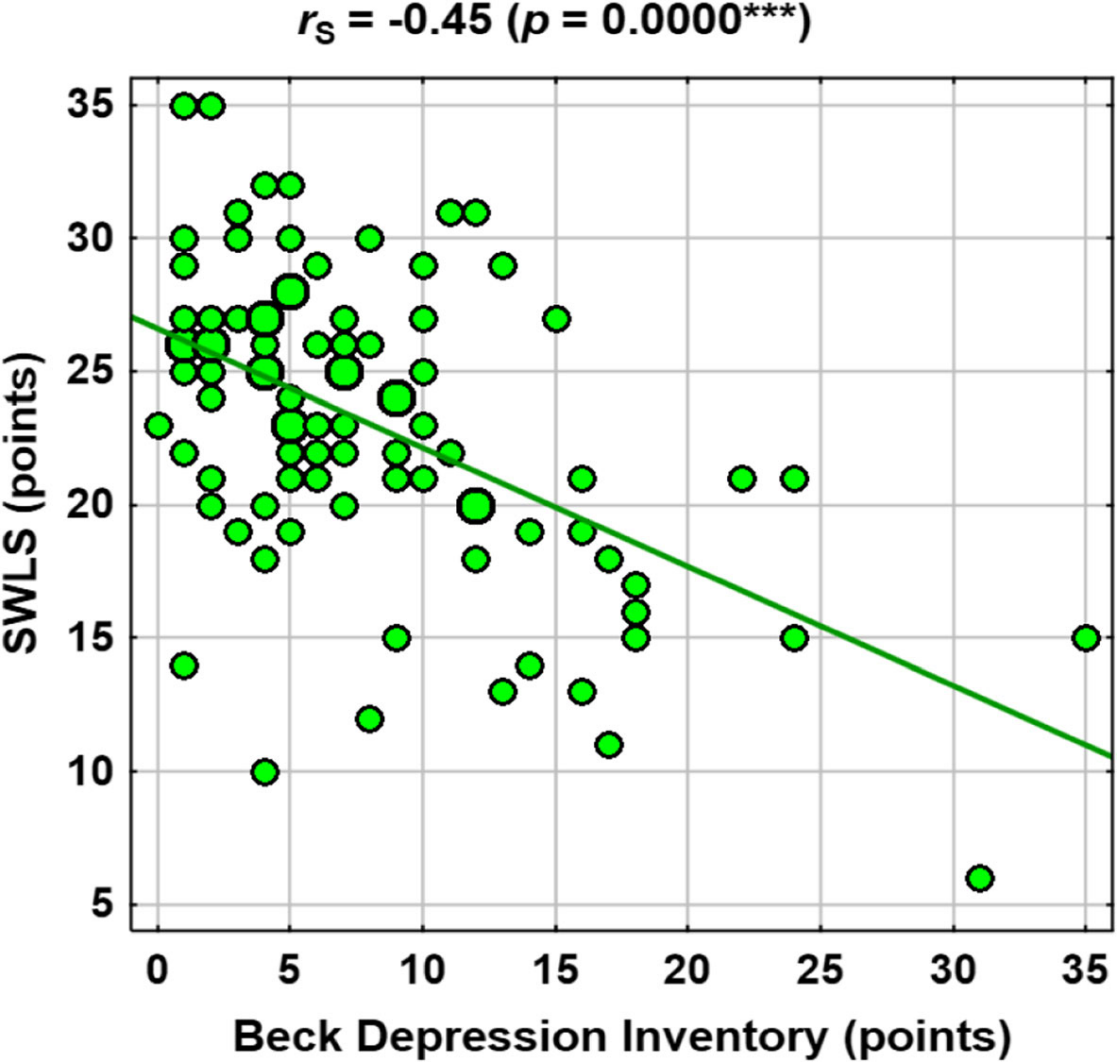
Correlations between BDI and SWLS

**Fig. 2:**
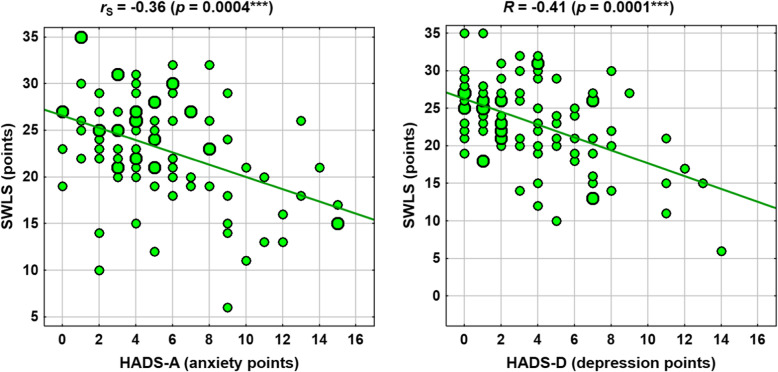
Correlations between HADS measures and SWLS

**Fig. 3:**
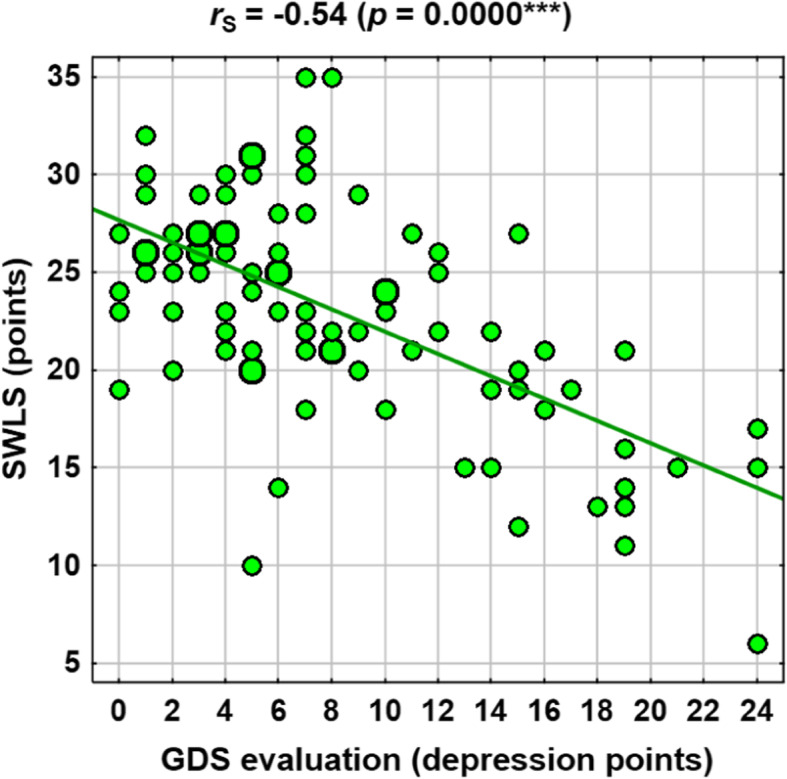
Correlations between GDS (depression points) and SWLS

**Fig. 4:**
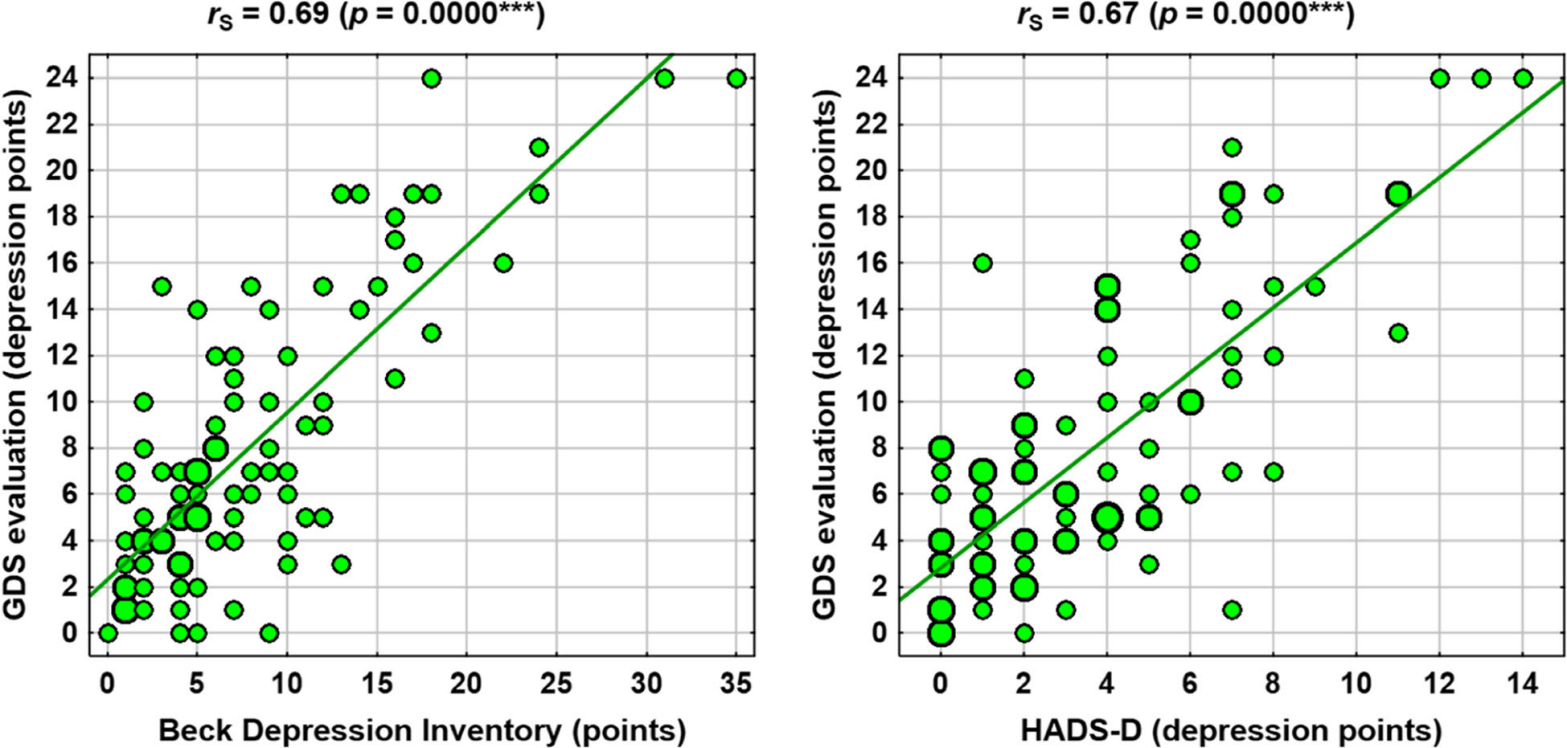
Correlations between depression scales (BDI vs. GDS and HADS-D vs. GDS)

Selected correlations are shown in the scatter plots in Figs. 1, 2, 3 and 4.

### Regression models for SWLS and GDS scores

The GDS was the measure of depression most strongly correlated with other measures of this type. Therefore, further analysis in this section was limited to a detailed analysis of the SWLS and GDS scores.

The results of the univariate analyses presented in Tables 4, 5, 6, 7 and 8 enable the identification of groups of older adults with relatively low life satisfaction and a higher level of depression: those who were single or in worse financial standing. To gain more complete insight into the relations between the factors considered in Tables 4, 5, 6, 7 and 8 and the life satisfaction and depression measure scores, regression analysis methods were used.

Regression models were constructed for the scores on the SWLS, a measure of life satisfaction, and the scores on the GDS, a measure of depression (as Table 9 shows, scores on this measure of depression were the most strongly correlated with the scores on the other measures).

The following set of explanatory variables was included in the analysis: sex, age, type of place of education, marital status and financial standing. Second-degree interactions between these factors were also considered. The GRM method was used for the analysis to search for statistically significant factors and interactions using the forward stepwise regression procedure. In the result tables, regression coefficient values (for both models with SWLS scores or GDS scores as dependent variables) were given only for the statistically significant factors.

**Fig. 5:**
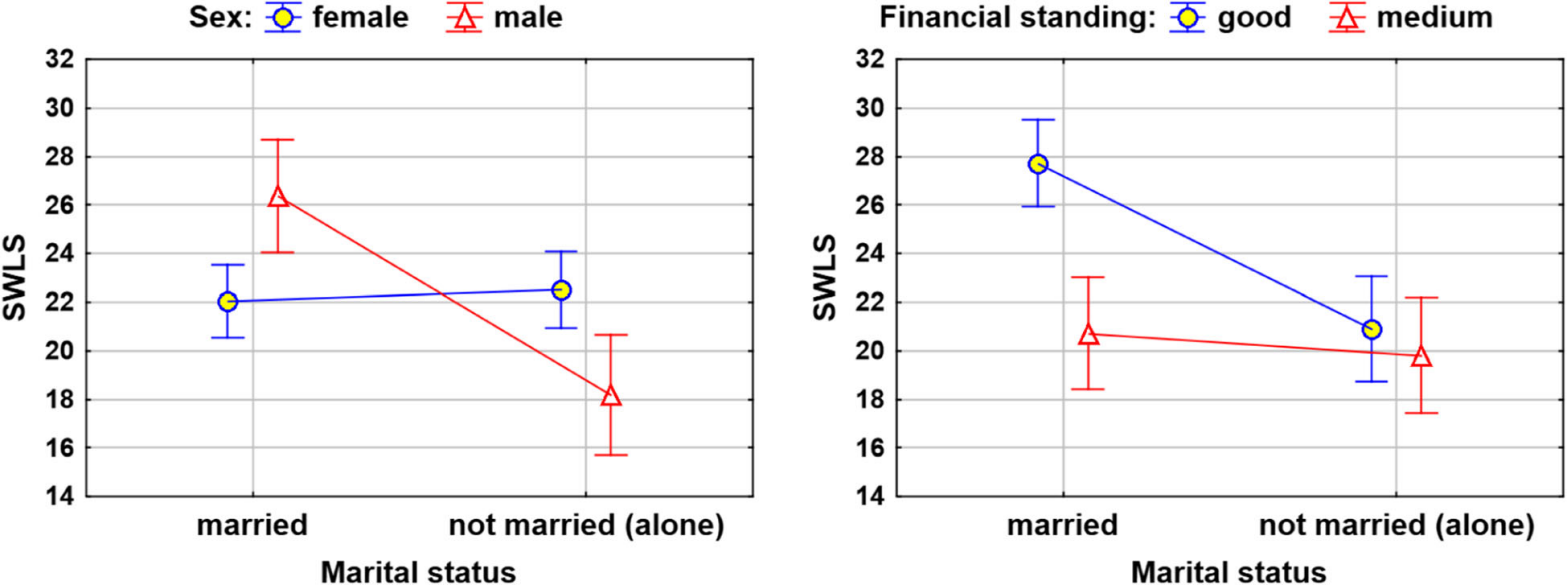
Interactions occurring in the regression model for SWLS (means with 95% CI)

To measure life satisfaction, four statistically significant factors were found. The model explained approximately 35% percent of the variability in life satisfaction (SWLS scores) in the surveyed population (Table 10). This is a relatively high result for analyses of psychometric measures (Fig. 5).

**Table 10:** Regression model for assessing SWLS

Independent factors	SWLS ***R***2 = 35.2% ***F*** = 11.4 ***p*** = 0.0000***		
	*B* (95% CI)		*β*
single person	−3.86 (−6.33; −1.39)	0.0025**	− 0.34
worse financial standing	−4.06 (−6.12; −1.99)	0.0002***	− 0.35
single person × sex (male vs female)	−4.34 (−6.80; −1.87)	0.0007***	−0.38
single person × worse financial standing	2.97 (0.91; 5.02)	0.0052**	0.26

*R*2 – coefficient of determination (the percentage of the response variable variation that is explained by a model); Test statistic *F* and *p*-value for assessment of significance of whole model

*B* – regression coefficient (with 95% confidence interval)

*p* – assessment of significance; *ß* – standardize regression coefficient

** *p* < 0.01, *** *p* < 0.001

**Fig. 6:**
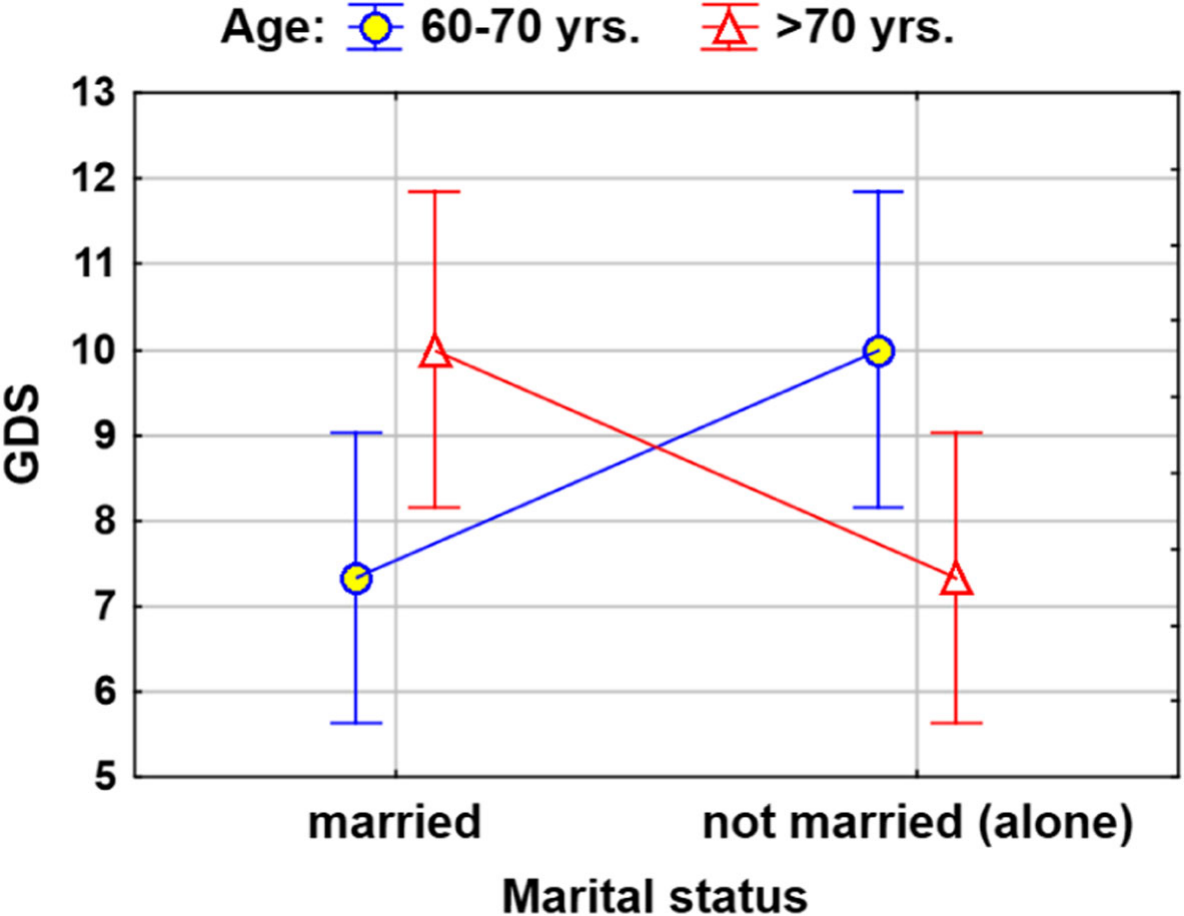
Interaction occurring in the regression model for GDS (means with 95% CI)

The regression model for the scores on the GDS, a measure of the severity of depression, identified much lower variability of this measure, only approximately 13% (Table 11), to be explained. Financial standing was a factor that significantly differentiates GDS scores; people who were less well-off had higher GDS scores by approximately 3.85 points on average. Moreover, there was a statistically significant interaction between age and being single. The nature of this interaction is described in the diagram (Fig. 6).

**Table 11:** Regression model for assessing GDS

Independent factors	GDS ***R***2 = 12.7% ***F*** = 6.3 ***p*** = 0.0029**		
	*B* (95% c.i.)	*p*	*β*
worse financial standing	3.85 (1.31; 6.39)	0.0035**	0.30
age above 70 × single person	−2.67 (−5.15; − 0.19)	0.0349*	−0.22

*R*2 – coefficient of determination (the percentage of the response variable variation that is explained by a model); Test statistic *F* and *p*-value for assessment of significance of whole model

*B* – regression coefficient (with 95% confidence interval)

*p* – assessment of significance; *ß* – standardize regression coefficient

** *p* < 0.01, *** *p* < 0.001

## Discussion

Depression is the second most frequent psychopathological syndrome among older adults and is considered one of the so-called “geriatric giants” [37]. Depression often occurs along with somatic problems, has various aetiologies and different clinical presentations, and significantly affects the deterioration of the quality of life and increased mortality in the older adult population [20, 21]. The prevalence of depressive symptoms in the population is higher than the prevalence of depression [38].

The analysis of studies on the incidence of depression among seniors shows a discrepancy in the results. This discrepancy may be related to a range of economic, health and social conditions in different countries. Our research has shown that an increased level of depression (according to BDI scores) occurs in less than one in four people. Similar assessments of the level of anxiety and depression were obtained on the basis of the HADS-A (26.4% with anxiety above the standard) and the GDS (28.9% with at least mild depression). The HADS-D assessments slightly differed from the other measures (in the classification according to this measure, only 13.2% of older adults showed symptoms of depression).

The authors’ results pertaining to the assessment of the incidence of depression show that the prevalence and risk of depression in the study group had intermediate values. In China, the incidence of depression was 3.86% [39], in Turkey it was 38.7% [40], and in Bangladesh it was 45% [41]. As the literature shows, worldwide, depression has an estimated average prevalence of 4.4%, with higher proportions for women (5.1%) than for men (3.6%) [42].

From a practical point of view, the issue of correlations between the assessments of the mental condition of older adults obtained on the basis of various questionnaires is important. This type of research should be conducted with due diligence and reliability. It is usually impossible to use several questionnaires because they are simply too tiring and time-consuming. Our research showed that all correlations between psychometric measure scores were statistically significant. The correlations between measure of life satisfaction (SWLS) scores and scores on other measures were obviously negative, and their strength was slightly smaller than the mutual correlations between scores on the measures of depression and anxiety. The GDS was the measure of depression for which the scores were most strongly correlated with the scores of other measures of this type. The results concerning the relationship between GDS scores and BDI scores are similar to those of other published reports in this field; for example, in the work by Jefferson et al. [43], the correlation between GDS scores and BDI scores was found to be 0.71, similar to the results presented in Table 5.

Snyder et al. noted, “The correlation between GDS and the BDI has also been found to be high (r=0.78) in a clinical sample of older adults” [44]. The studies conducted by Leś et al. [45], in which no statistically significant correlations between age and mood dimensions were found, confirm our results: the mental condition of older adults did not differ on the basis of age nor place of education, and age was not associated with differences in depression levels.

Age was not associated with mental health status. It can be presumed that social and mental activity inhibits the negative association of age with mental condition. Recent studies have shown that participation in prosocial activities, such as volunteering, predict better mental and physical health in late adulthood and that participation in prosocial activities may protect against the negative ramifications associated with older age [46]. Similar conclusions were drawn by Cybulski et al. [47] and Segal et al. [48].

In the PolSenior study, in which the Geriatric Depression Scale (GDS) was used, it was noted that the frequency of depressive disorders among seniors in Poland increased with age (20% for the 55–59 age group, 25% for the 65–79 age group, 33% for 80 and older) [49]. Similar results were presented by Weterle and Sołtysiak [50] and a team of German researchers [51]. Different results were obtained in a study conducted among Germans aged 53–80, where depression was the most common in the youngest age group [52].

A number of studies show that age, combined with other psychosocial factors and the incidence of somatic diseases, can become a risk factor for depression [24, 53–55].

The prevalence of depression among seniors is estimated to be between 10 and 20% [56]. In Poland, in the WOBASZ study, based on Beck’s Depression Scale, the occurrence of depressive symptoms in the general Polish population was estimated at approximately 25% [55].

In our research, which was conducted using the GDS, no depression was found in over two-thirds of respondents.

A low level of depression among surveyed seniors was found in a study conducted by Leś et al. [45] using the same scale with 116 attendees (women) of the University of the Third Age. The results showed that only approximately 6% of respondents revealed symptoms of depression, mainly moderate symptoms [45]. Other authors indicate a greater problem of depression among the general population of surveyed seniors of 41.72% [50].

In our study, it was observed that the sex of respondents was not correlated with depression levels and that depression levels did not differ on the basis of sex.

This result was also found by Cybulski et al. [47] and Segal et al. [48]. Other conclusions were reached by Weterle and Sołtysiak, who observed that women had higher levels of depression in the examined group of primary care patients [50]. In the international literature, other studies confirm that older women are more vulnerable to depression [51, 57–59]. Studies conducted by Norwegian researchers have shown that the prevalence of self-diagnosed depression in the adult Norwegian population was higher for women than for men [42].

As indicated in this paper, it is worth mentioning recent studies in which the authors noted differences regarding the impact on scientific literature on active ageing, life satisfaction and the quality of life of older adults between European projects as part of the Seventh Framework Programme and similar US projects funded by the National Institutes of Health (NIH) [2, 3]. Carta et al. [2] showed that, with regard to scientific publications, the European Union maintained its excellence in production in terms of both quality and quantity. However, the EU states lost their position, particularly in some of the emerging fields of science, including biomedicine. In 2017, only Germany, Italy and Poland maintained their SCIMAGO ranking for scientific production achieved 10 years earlier (fourth, eighth and eighteenth, respectively), while other states, such as France, Spain, the Netherlands, Sweden, Belgium and Austria, moved down in this ranking. The authors of the study noted that among the top 100 research centres worldwide, the number of European centres dropped from 14 to only 10 in 2018. The study indicated lower cost-effectiveness of the FP7 European projects than of the American NIH projects with regard to active ageing. This inferior result was established only in relation to the cost of the researchers, while the impact on the literature was equal. According to the researchers, differences between the landscape of research and innovation in the EU and the US, as well as between their policies, may determine the difference in the project costs; however, the results of this research with consideration of their limits will have to be examined in future evaluative studies [2].

An observational evaluative study by Kirilov et al. [3] aimed to verify whether European projects on Active Ageing (AA) and Elderly Quality of Life (QoL) funded by the Seventh Framework Programme (7PR) had a similar impact on the international literature as US projects funded by the National Institutes of Health (NIH) using well-known bibliometric indicators. The study found that the results of active ageing projects financed by the EU had an impact on international scientific literature and a level of visibility comparable with that of similar projects financed in the US by the NIH [3]. The authors of the study believe that their efforts may be useful in developing standard and repeatable procedures [3].

The study showed that single people were characterised as having lower life satisfaction than married people by approximately 3.86 points on average, similar to people in worse financial standing (who had an SWLS score that was lower by 4.06 points).

Interesting interactions were found: single men reported lower quality of life, while in the case of single women, quality of life tended to be higher. Financial standing was associated with the life satisfaction of married people, while for single people, this issue was not of great importance. Research by Han et al. showed a significant relationship between marital status and quality of life (QOL), and this relationship appeared to differ on the basis of sex and age; however, the regression coefficients for divorced men or women were not statistically significant [60]. On the other hand, studies by Gutiérrez-Vega et al. [61] indicated that with regard to the health component of quality of life, single and married older adults had higher scores than widowed and divorced adults; similarly, married older adults had the highest quality of life in terms of social relationships. The authors of the study suggest that marital status may play an important part in the analysis of quality of life among older adults. The study also suggests that being married may offer a protective mechanism against depressive symptoms and therefore against mental illnesses during late adulthood [61].

Furthermore, our research showed that single people were less depressed in the group aged over 70, while in the group aged 60–70, the opposite was true. Studies by Seddigh et al. indicated that older adults in day centres showed the lowest levels of depression, while those living in nursing homes had the highest levels of depression [62].

Our study found that older men were characterised by a slightly higher satisfaction with life, and the difference was close to statistically significant. Depression and anxiety measures did not differ on the basis of sex.

Similar results were obtained in studies on life satisfaction and depression among Japanese seniors [63]. A similarly negative correlation between the severity of depressive symptoms and all indicators of quality of life was observed among students of the University of the Third Age [45]. Daly et al. showed in their studies that depressive disorders had a negative effect on the sense of quality of life [18]. The studies conducted by Fiske et al. [64] also demonstrated that women with a low level of life satisfaction were more prone to depression.

The feeling of life satisfaction is related to various factors, including cultural factors. Due to different cultural contexts, caution should be used when comparing life satisfaction between different countries [65]. Earlier research conducted among students of the University of the Third Age in Poland showed that the vast majority (75.2%) were satisfied with their lives [66].

The results of our study are based on a community of university students who were pensioners and were not directly in the depression risk group, which involves factors such as a reduction in intellectual skills, infrequent participation in social activities and reduced interpersonal relationships [67, 68]. The results of this study are consistent with those of earlier studies conducted in various cultural circles, confirming the positive association of participation in such activities with life satisfaction, self-esteem and depression level [69–71].

It is worth emphasising that the lack of differences in the level of depression between people of different age groups, as shown in this study, may indicate the prevention of the negative effects of the ageing process by being socially and mentally active.

In Polish society, there is an increasing number of older adults, and maintaining social, professional and family activities as long as possible depends on good habits, behaviour and an active lifestyle. The Ministry of Health supported the implementation of a care programme for the prevention of the negative effects of the ageing process through activities as part of the National Health Programme 2016–2020, which included educational activities and adapting the health care system to the needs of older adults [72]. As this research [73–76] shows, it can be assumed that older adults who are physically and mentally active will require such care later in their life.

### Limitations

The study presented here is not representative of the entire senior population in Poland. The examined attendees of the University of Healthy Senior and auditing students of the University of Psychogeriatric Prophylaxis are better educated and more intellectually active and come from larger cities than their peers in the older adult population. One limitation of the study was the small size of the study group and the lack of a control group. Furthermore, the study was conducted in one city only (Bialystok), which may not reflect the results for the entire country. Another limitation was the group selection, as there is a risk that the community of university students who are pensioners was less appropriate than the whole senior population in Poland. This group may not have been the best choice for a true representation of the entire population of the region, as they likely had better physical health and may have been wealthier than other members of the community.

## Conclusions

The high level of life satisfaction and the low level of depression should be maintained in the surveyed group of mentally active people living in the Voivodeship of Podlaskie in Poland, and additional educational activities should be organised among seniors on a large scale. There were no differences in the distribution of psychometric measure scores among the three compared age groups of respondents in this study. The correlations between the scales used do not seem to be high enough to replace one questionnaire with another without losing information. The lack of very strong correlations indicates that each of the questionnaires measures different aspects of depression, and we believe that it is worth using them in parallel rather than interchangeably.

### Practical aspects of the study

The results of the analysis investigating the correlations between the point scales for assessing the level of depression and anxiety, determined by means of different questionnaires, are of practical importance. If there were strong correlations, it could be concluded that one of the questionnaires from which information about the severity of depression in a given community was revealed by could be replaced by another questionnaire, which would shorten and facilitate the study.

## Data Availability

Data supporting the findings are available upon request. Please contact the corresponding author, Katarzyna Van Damme-Ostapowicz (katarzyna.van.damme-ostapowicz@hvl.no), for data access.

## Abbreviations

UHS: University of Healthy Senior
UPP: University of Psychogeriatric Prophylaxis
SWLS: Satisfaction with Life Scale
BDI: Beck Depression Inventory
GDS: Geriatric Depression Scale
HADS: Hospital Anxiety and Depression Scale
